# Effects of *β*-Cryptoxanthin on Improvement in Osteoporosis Risk: A Systematic Review and Meta-Analysis of Observational Studies

**DOI:** 10.3390/foods10020296

**Published:** 2021-02-02

**Authors:** Sun Jo Kim, Nguyen Hoang Anh, Nguyen Co Diem, Seongoh Park, Young Hyun Cho, Nguyen Phuoc Long, In Guk Hwang, Johan Lim, Sung Won Kwon

**Affiliations:** 1 College of Pharmacy, Seoul National University, Seoul 08826, Korea; danielkim27@snu.ac.kr (S.J.K.); 2018-23140@snu.ac.kr (N.H.A.); 2 School of Medicine, Vietnam National University, Ho Chi Minh City 70000, Vietnam; ncdiem.stu15@medvnu.edu.vn; 3 Department of Statistics, Sungshin Women’s University, Seoul 02844, Korea; spark6@sungshin.ac.kr; 4 Department of Statistics, Seoul National University, Seoul 08826, Korea; yhjo93@snu.ac.kr (Y.H.C.); johanlim@snu.ac.kr (J.L.); 5 Research Institute of Pharmaceutical Sciences, Seoul National University, Seoul 08826, Korea; phuoclong@snu.ac.kr; 6 Researcher, Department of Agrofood Resources, National Institute of Agricultural Sciences, Rural Development Administration, Wanju 55365, Korea; ighwang79@korea.kr

**Keywords:** *β*-cryptoxanthin, osteoporosis, bone mineral density, hip fracture, carotenoid, clinical study, systematic review, and meta-analysis

## Abstract

Many studies have analyzed the effects of *β*-cryptoxanthin (BCX) on osteoporosis and bone health. This systematic review and meta-analysis aimed at providing quantitative evidence for the effects of BCX on osteoporosis. Publications were selected and retrieved from three databases and carefully screened to evaluate their eligibility. Data from the final 15 eligible studies were extracted and uniformly summarized. Among the 15 studies, seven including 100,496 individuals provided information for the meta-analysis. A random effects model was applied to integrate the odds ratio (OR) to compare the risk of osteoporosis and osteoporosis-related complications between the groups with high and low intake of BCX. A high intake of BCX was significantly correlated with a reduced risk of osteoporosis (OR = 0.79, 95% confidence interval (CI) 0.70–0.90, *p* = 0.0002). The results remained significant when patients were stratified into male and female subgroups as well as Western and Asian cohorts. A high intake of BCX was also negatively associated with the incidence of hip fracture (OR = 0.71, 95% CI 0.54–0.94, *p* = 0.02). The results indicate that BCX intake potentially reduces the risk of osteoporosis and hip fracture. Further longitudinal studies are needed to validate the causality of current findings.

## 1. Introduction

Osteoporosis is a chronic and systematic skeletal disease characterized by reduced bone mineral density (BMD), eventually leading to bone fragility and bone fracture [1]. Hip fracture, a prevalent complication associated with low hip BMD, is positively associated with osteoporosis, especially in the elderly population [2]. Notably, several reports have stated that nutrients including calcium, vitamin D, and carotenoids-enriched fruits and vegetables and dietary regulations can reduce osteoporosis risk [3,4,5].

Beta-cryptoxanthin (BCX) belongs to a group of carotenoids that are naturally occurring pigments. In particular, BCX is well known to be abundant in citrus fruits such as orange and mandarin [6]. It exhibits vitamin A activity because its structure contains a beta-end group similar to *β*-carotene. Similar to other carotenoids, BCX is susceptible to oxidative and enzymatic cleavage, which subsequently forms many bioactive metabolites [7]. Various potential health effects have been previously described, including cancer prevention, antioxidation, and osteoporosis reduction, just to name a few [6,8,9]. Therefore, many studies exploring the health efficacy of BCX have been routinely reported in recent years, including laboratory works and clinical research. Accordingly, evidence from epidemiological studies has proposed various beneficial effects against cancers, osteoporosis [10,11,12,13], and all-cause mortality [12].

A certain number of clinical studies have recently investigated the association between the dietary pattern of BCX and osteoporosis risk [14,15,16]. In addition, BCX in human blood was also examined to evaluate the correlation [17,18]. A promising correlation was observed in terms of reduced risk of osteoporosis and bone fractures, as they are reported. However, the findings are not consistent among studies, and more importantly, there is a lack of quantitative evidence and a comprehensive review of the effect of BCX on bone health. Therefore, we conducted a systematic review and meta-analysis to reveal the association of BCX with osteoporosis and common bone fractures. Moreover, an overview of the current status of BCX-related research was further discussed.

## 2. Materials and Methods

All the steps from literature search to data synthesis and quality assessment were collaboratively performed by at least two well-disciplined reviewers. Our study followed the guidelines of Meta-analyses Of Observational Studies in Epidemiology (MOOSE) Checklist (Table S1) [19].

### 2.1. Literature Search and Selection Criteria

Studies were collected by searching on international literature databases including PubMed, Embase, and Cochrane Central Register of Controlled Trials (CENTRAL) with the following search terms: (“*β*-cryptoxanthin” or “beta-cryptoxanthin” or “cryptoxanthin” or “cryptoxanthine” or “beta-caroten-3-ol” or “carotene-3-ol” or “carotenol”) and (“osteoporosis” or “osteoporotic” OR “osteopenia” or “osteoblast” or “osteoclast” or “bone”). The search was conducted on the first day of July and was not limited to their publication dates. All types of clinical observational studies investigating BCX with regard to osteoporotic-related health problems were defined as eligible. After being imported into Endnote X9, duplicates were screened and filtered. The remaining studies were screened for their titles, abstracts, and keywords to examine their eligibility. A study was excluded for the following reasons: (1) the inclusion criteria were violated, (2) only irrelevant data was presented to extract, (3) the topic was unrelated (4) it was not a peer-reviewed research article, (5) it was duplicated entirely in both cohort and subject with another study, or (6) the full text was unavailable. Manual screening on the reference list of previous articles was conducted, and periodic checks were carried out after the search date to complement additional studies.

### 2.2. Data Extraction

After careful evaluation by two independent authors, eligible studies were included for data extraction and meta-analysis. All relevant information on individual research was collected, including authors, publication year, cohort allocation, population characteristics (e.g., sex and age), and sample size (e.g., the population of case and control groups). Regarding outcome measurement, data of evaluation parameters, statistical models, adjusted factors for statistical analysis, and primary outcomes were extracted.

### 2.3. Data Synthesis

We primarily conducted a meta-analysis to evaluate the overall association between osteoporosis-related outcomes and BCX intake. We included only studies that estimated the association of osteoporotic risk-related outcomes reported as odds ratio (OR) and 95% confidence interval (95% CI). Studies comparing bone-related risk between the highest-level and the lowest-level groups (e.g., quintile 5 versus quintile 1, quartile 4 versus quartile 1, or tertile 3 versus tertile 1) were included in the pooled analyses. When multiple correlation models were reported from a single cohort, the adjusted model that encompassed the most confounding variables was used. If a study did not provide an adjusted OR, imputation was applied by calculating the raw OR using the provided data. Owing to the lack of complete information from the study of Sahni et al., ORs from the previously published meta-analysis were employed in the current data synthesis [20].

The data were synthesized using RevMan 5 (the Cochrane Collaboration). ORs from each publication were pooled using the generic inverse variance method with the random-effects analytic model. Heterogeneity across the studies was reported using the Cochran Q test and *I*^2^ statistic. A funnel plot was not applied in this study as well as other analyses of publication bias due to the small number of included studies.

### 2.4. Sensitivity Analysis

Leave-one-out sensitivity analysis was conducted to inspect the robustness of the metadata. Sensitivity analysis was performed for all the outcomes of the meta-analysis, including pooled ORs of the overall osteoporotic risk and its subgroups.

### 2.5. Quality Assessment

Methodological quality was evaluated based on the study types using the tools from the National Health Institute (NIH). Critical study design-specific aspects related to the risk of bias were independently inspected for the included studies. Each item was scored as “Yes” or “No” according to the qualification. Otherwise, they were classified as “cannot determine (CD)”, “not applicable (NA)”, or “not reported (NR)” depending on the information provided. After evaluation, each author provided an overall quality rating for each study. The conflict was solved by the third reviewer’s arbitration.

## 3. Results

### 3.1. Literature Selection

**Figure 1:**
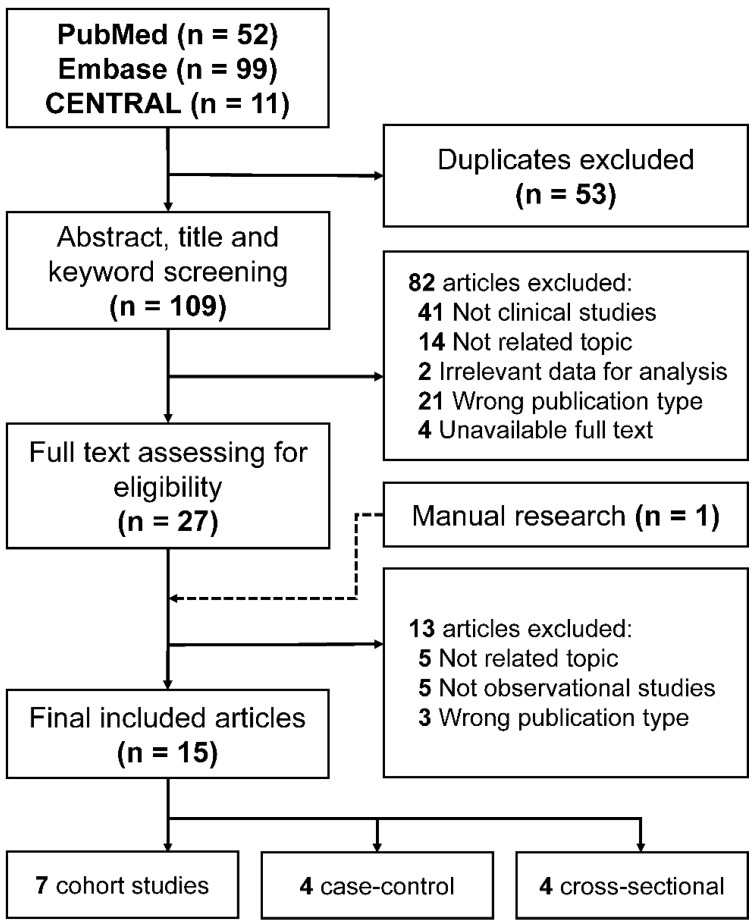
Flowchart of literature selection process.

The overall workflow of the current systematic review and meta-analysis is presented in Figure 1. After a comprehensive search of the databases, we collected 52, 99, and 11 articles from PubMed, Embase, and CENTRAL, respectively. One hundred and nine studies were subsequently screened for their title, abstract, and keywords to assess their eligibility after removing duplicates. Next, 27 studies were subjected to full-text investigation. Finally, 15 observational clinical studies were included for data extraction, including one manually added research. Among 15 observational studies, seven provided data of ORs in their reports.

### 3.2. Methodological Quality

The methodological qualities of all studies were rated as “fair” to “good” (Table 1). However, there were still potential risks of bias due to the lack of sample size justification, multiple outcome measurements, and the blinding of the investigator. Complete evaluation results are described in Tables S2 and S3.

### 3.3. Meta-Analysis of the Effects of β-Cryptoxanthin on Osteoporosis

The detailed demographic characteristics, evaluation parameters, and outcomes are described in Table 1 and Table S4. Regarding the study design, BCX was examined in seven cohort studies, four case-control studies, and four cross-sectional studies. The number of populations in each study ranged from 59 to 25,566. The degree of BCX exposure was estimated by the amount of BCX intake through the food intake questionnaire, as well as the BCX serum level. The differences in the amounts of BCX intake were described as the fractiles in all related studies. BMD was the most frequently evaluated risk parameter, followed by the incidence of fractures and others.

**Figure 2:**
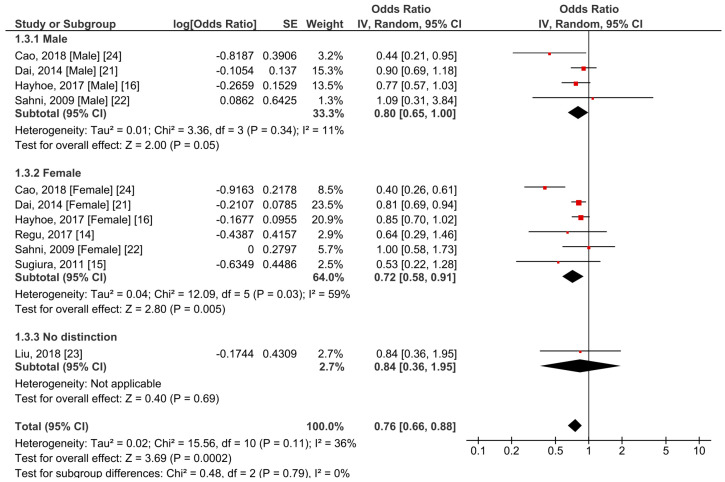
Meta-analysis results of *β*-cryptoxanthin (BCX) intake and osteoporosis-related outcome in male and female subgroups.

Seven studies including 100,496 participants were included in the meta-analysis [14,15,16,21,22,23,24]. Of note, four studies overlapped the investigated cohort [15,26,27,30]; therefore, only one with the highest quality was included in the meta-analysis [15]. We found a significant negative correlation between the overall risk of osteoporosis and the amount of BCX intake with an OR of 0.76 (95% CI 0.66–0.88, *p* = 0.0002) (Figure 2). The overall heterogeneity between the studies was 36% (*p* = 0.11). Due to the physiological differences between males and females, subgroup analysis stratified by sex was conducted. As a result, we observed that the high intake of BCX in female subjects was significantly correlated with a lower risk of osteoporosis than that in the lower consumption group (OR = 0.72, 95% CI 0.58–0.91, *p* = 0.005, *I*^2^ = 59%). A similar result was observed in male subjects but to a smaller extent of reduced osteoporotic risk (OR = 0.80, 95% CI 0.65–1.00, *p* = 0.05). The heterogeneity in the male subgroup population was relatively low (*I*^2^ = 11%). In addition, there was no significant heterogeneity between the male and female subgroups (*p* = 0.79).

**Figure 3:**
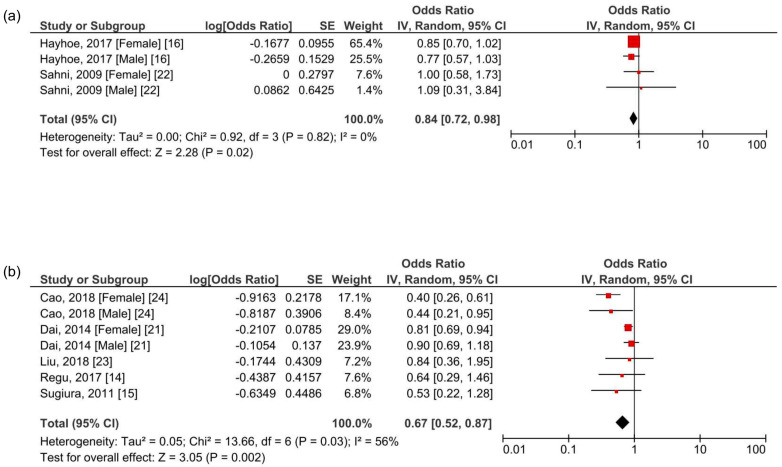
Meta-analysis results of BCX intake and osteoporosis-related outcome by ethnicity. (**a**) Western (the United Kingdom and the United States); (**b**) Asia (Singapore, Korea, Japan, and China).

In a further subgroup investigation, the correlation between BCX intake and osteoporosis was stratified by ethnic characteristics. A meta-analysis of the Western population that included the United Kingdom and the United States revealed a significantly reduced osteoporotic risk in a high BCX intake population with a pooled OR of 0.84 (95% CI 0.72–0.98, *p* = 0.02, *I*^2^ = 0%) (Figure 3a). Asian populations including Singapore, Korea, Japan, and China showed a similar significant negative correlation with a pooled OR of 0.67 (95% CI 0.52–0.87, *p* = 0.002, *I*^2^ = 56%) (Figure 3b).

**Figure 4:**
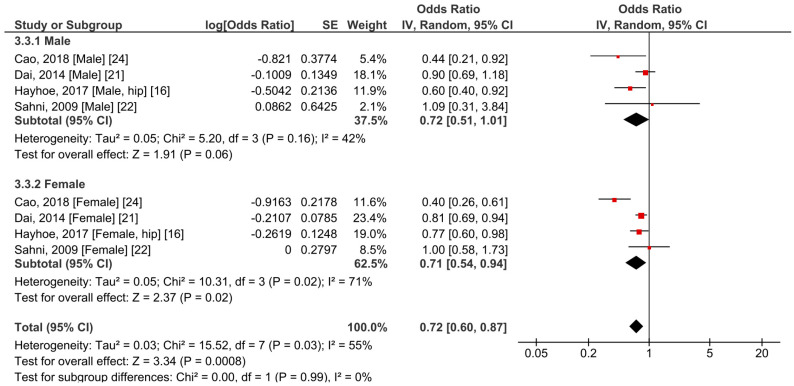
Meta-analysis results of BCX intake and hip fracture in males and females.

Because hip fracture is one of the most common complications of osteoporosis, we additionally conducted a meta-analysis focusing on this particular fracture position. In total, four studies recruiting 76,663 participants provided sufficient information for the meta-analysis. All included studies explored the outcome of both sexes, with ages ranging from 39 to 80 years. Therefore, a subgroup analysis based on sex was also conducted. Overall, the results revealed that a higher BCX intake was significantly correlated with fewer events of hip fracture (OR = 0.72, 95% CI 0.60–0.87, *p* = 0.0008, *I*^2^ = 55%) (Figure 4). Considering only the male subjects, a trend toward a lower risk of hip fracture was observed, although without significant difference (OR = 0.72, 95% CI 0.51–1.01, *p* = 0.06, *I*^2^ = 42%). A higher intake of BCX was also significantly correlated with a lower risk of hip fracture in the female subgroup (OR = 0.71, 95% CI 0.54–0.94, *p* = 0.02, *I*^2^ = 71%). No significant difference was found between the groups (*p* = 0.99).

### 3.4. Sensitivity Analysis

Male and Western ethnic subgroups, based on the overall risk of osteoporosis, were susceptible to changes according to the leave-one-out sensitivity analysis. Meanwhile, both male and female subgroups were also sensitively affected in terms of the outcome of the risk of hip fracture. In all cases, elimination of the data from Cao et al. remarkably reduced the heterogeneity.

### 3.5. Other Observational Studies

We recorded eight other studies that reported an association between BCX and the incidence of osteoporosis. However, no data were available or suitable for conducting the meta-analysis. Of note, all eight studies investigated the association between blood BCX and osteoporotic risk. A summary of characteristics and key findings is given in Table 1**.**

## 4. Discussion

Previous studies have suggested notable beneficial functions of BCX. Among them, the specific benefits of the effects of BCX on bone homeostasis have received more attention in recent years, particularly in clinical studies. Our primary result of the meta-analysis was that a high amount of dietary BCX was significantly correlated with a low risk of osteoporosis. In addition, corresponding outcomes were also drawn from the subgroup analyses. Females benefited more than males when they consumed a high amount of BCX. Another interesting finding was that a consistent and significant relationship was observed in different cohort ethnicity. However, due to the limited number of studies, the confidence and robustness of the results need to be further validated with quality control procedures by sensitivity analysis.

Numerous studies have investigated natural products and compounds to discover their efficacy on osteoporosis [33,34,35]. It was reported that a diet rich in fruits and vegetables was positively associated with a reduced risk of osteoporosis and fracture in both sexes, after conducting a systematic review and meta-analysis on the effect of dietary patterns on BMD value and fracture risk [36,37]. BCX, which is one of the major carotenoids present in various fruits such as citrus (e.g., 407 µg of BCX/100 g of tangerines), persimmons (1447 µg of BCX/100 g), and papaya (589 µg of BCX/100 g), has been focused on its beneficial role for maintaining bone homeostasis [6,38,39]. For example, an oral 10 mg/L BCX supplementation in drinking water significantly prevented bone loss in an ovariectomized mice model after 28 consecutive days of treatment [40]. A previous study revealed that exposure to BCX for two to three days stimulated proliferation of osteoblastic cells proliferation provoked transcription of insulin-like growth factor-1 and transforming growth factor-β, which are bone growth factors [41]. Exposure to BCX for three days induced mRNA expression of cell differentiation and mineralization factors including Runx2, type I collagen, and alkaline phosphatase in osteoblastic MC3T3-E1 cells [42]. This aligns with the finding that 10 µM of all-trans retinoic acid promotes osteogenic differentiating genes and angiogenic genes in rat bone marrow-derived mesenchymal stem cells [43]. Another interpretative mechanism is that BCX induces the activation of osteoblasts by downregulating NF-κB activation [44,45]. NF-κB also plays a role in the regulation of osteoclasts modulated by nuclear factor kappa-Β ligand (RANKL) and tumor necrosis factor-α [46]. Therefore, BCX is expected to repress the proliferation of osteoclasts. It was confirmed in a previous investigation on RAW264.7 cells that BCX directly suppressed both RANKL and inhibitor of NF-κB kinase [47]. Corresponding to the aforementioned findings, several interventional clinical studies have attempted to observe the response of bone turnover markers to BCX intake [17,18,48,49,50]. Except for a study conducted in the UK wherein the researcher could not find evidence between higher serum BCX concentration and bone turnover markers, four interventional studies reported a positive correlation between BCX intake and osteoporosis risk. However, there was a disagreement in the tendency of osteocalcin alteration among studies, demanding that further studies on bone turnover markers are required to arrive at a consensus.

The physiological aspects of osteoporotic risk depend on sex, particularly in postmenopausal women. Therefore, a daily diet with sufficient vitamins, calcium, and carotenoids is particularly crucial to postmenopausal women [3]. Our findings emphasize the important relationship between a diet rich in BCX and the risk of osteoporosis and fractures in pre- and postmenopausal women [3]. The effect on the risk of postmenopausal osteoporosis was once again confirmed in another meta-analysis on observational studies, wherein high consumption of vegetables and fruits significantly reduced osteoporosis risk [51]. In addition, an ethnicity-specific response was also observed in our results. Previous studies have mentioned that the association between nutrition and bone health varied depending on ethnicity [3,52]. The present study displayed an agreement with previous study findings by showing the difference in the extent of ORs regarding the population demographics. In detail, a higher response to BCX intake was recognized in Asian subjects than in Western subjects.

Hip fracture, the most common osteoporosis-related complication, is a significant health problem leading to a decrease in life expectancy, disability, and mortality in the elderly population [53]. In this study, we found that the risk was correspondingly lower when we specifically focused on the risk of overall hip fracture. Intriguingly, the result showed a contrasting trend to the previous publications, where the authors reported an increased but insignificant tendency on hip fracture risk [20]. Significant effects were also observed in both male and female subgroups, with a slightly greater extent for females. Similar to the outcome from the overall risk of osteoporosis, we confirmed again that the risk of hip fracture upon BCX intake was more sensitively affected in female subjects. Moreover, we updated the previous meta-analysis on the association between BCX intake and hip fracture risk [20] to significantly improve aspects. However, the moderate heterogeneity of the total and subgroup analyses could not be neglected, as well as the susceptibility of each subgroup to the sensitivity analysis. Therefore, the outcome should be interpreted with caution.

In addition to the investigation of BCX intake, the correlation of serum BCX levels was also commonly explored. Considering that Sugiura et al. performed statistical analysis on the same cohort, four cohort populations were analyzed to investigate the correlation between BMD and BCX intake or serum BCX level [14,15,25,26,27,30]. In general, exposure to BCX was positively associated with high BMD in the forearm, femur neck, lumbar spine, and hip. Noticeably, all improvements were observed in postmenopausal women residents in Asian countries. These results further support the potential ethnicity-based difference in the basal BMD level between the Western and Eastern populations, which is also presented in our meta-analysis results. Changes in BMD can be evident in Asian subjects, as they generally show a low BMD level [54,55]. In contrast to these qualitative findings, subgroup analysis of the metadata revealed the alleviation of osteoporotic risk in both Western and Asian populations. However, further studies should be conducted to confirm the precise efficacy on BMD, because the number of studies was insufficient, and different fractions of BCX intake were compared. It is also important to note that methodological description of selective analysis on blood BCX as compared with its structural isomer, α-cryptoxanthin, was insufficient in most of the studies, although it is generally a hurdle to separate them in chromatographic condition. Considering the fact that these isomers generally coexist in plant matrices, a potential contribution of α-cryptoxanthin on the reduction of osteoporosis risk cannot be ruled out from meta-analysis result, and therefore further interventional studies on each component are needed.

This study is the first systematic review and meta-analysis of the association between BCX intake and the overall risk of osteoporosis. Sex and ethnicity were also associated with the degree of risk reduction. Meanwhile, the result is an updated meta-analysis on the risk of hip fracture. There are several limitations to this systematic review and meta-analysis, which should be specified in the current investigation. First, the number of quantitatively synthesized studies was small, as only seven out of 15 studies were incorporated into the meta-analysis because of the impractical format of data, such as the beta coefficient of the regression model, overlapping cohort, or lack of relevant data. Second, imputation of the unavailable data might introduce an unexpected error in the data synthesis procedure. Third, another potential source of bias was the heterogeneity of study design across the included studies, as well as the comparison by combining either different fractiles of BCX intake or differently adjusted covariates. Likewise, outcome variables could not be matched due to the inconsistency in outcome measurements. The estimation of BCX intake, in the included studies, was difficult to clarify because of limited access to individual food questionnaires. Finally, multiple cross-sectional and case-control studies were involved, but the causality of the exposure and the event was not reported. Taken collectively, the current meta-analysis supports the previous idea regarding the potential benefit of BCX intake in lowering the risk of osteoporosis and fracture. Further investigations with a solid study design and consistent outcomes should be conducted.

## 5. Conclusions

In conclusion, the evidence of this meta-analysis suggested that the intake of BCX was significantly associated with a lower risk of osteoporosis. Consistent trends were observed in the male and female subgroups and both Western and Asian cohorts. The risk of hip fracture was also negatively associated with BCX intake. Moreover, 15 observational studies that investigated the effect of BCX on osteoporotic risk were summarized with a detailed description of study characteristics. Collectively, our findings can guide the development of the functional use of BCX. Further longitudinal studies and randomized controlled trials on this aspect are required to address a higher level of evidence.

## Figures and Tables

**Table 1:** Demographic characteristics, evaluation parameters, and outcomes of 15 observational studies.

Author, Year, Location	Included to Meta-Analysis	Study Design	Total Population (Age as a Range or Mean ± SD)	Sex	BCX Exposure	Outcome Variables	Bias Variables Adjustment	Summary	Quality Rating ^†^ (Risk of Bias)
Regu, 2017, South Korea [14]	Yes	Cross-sectional	8022 (age 30–74)	Male, pre-/postmenopausal female	Quintile of BCX intake (postmenopausal female): Q1 (0.0003–0.01 mg/day) Q2 (0.01–0.04 mg/day) Q3 (0.04–0.10 mg/day) Q4 (0.10–0.59 mg/day) Q5 (0.60–18.53 mg/day)	Coefficient of BCX intake with the BMDs of femur neck, total hip, lumber spine, and whole body; odds ratios for femur neck, total hip, lumber spine, and total osteopenia.	Yes	On pre-menopausal women, BCX consumption was positively correlated with femur neck BMD and total hip BMD. BCX consumption was also positively correlated with total hip BMD.	Fair
Sugiura, 2011, Japan [15]	Yes	Cross-sectional	293 (age 60.2 ± 6.2)	Postmenopausal female	Tertile of BCX intake Q1 (0.00–0.30 mg/day) Q2 (0.31–1.21 mg/day) Q3 (1.22–7.91 mg/day)	Odds ratios for BMD	Yes	Higher daily intake of BCX significantly prevented the risk of osteoporosis.	Fair
Hayhoe, 2017, United Kingdom [16]	Yes	Prospective cohort (12.5 mean years)	Fracture analysis data, 25,566; ultrasound analysis data, 14,877 (age 39–79)	Male, pre-/postmenopausal female	Quintile of BCX intake, quintile of serum BCX concentration	Calcaneal broadband ultrasound attenuation (BUA); total fracture; hip fracture; spine fracture; wrist fracture	Yes	The amount of BCX intake is positively related to the higher BUA value in women. In men, the hip fracture risk was significantly lower in the 5th quintile as compared with the 1st quintile with a 0.65 hazard ratio.	Good
Dai, 2014, Singapore [21]	Yes	Prospective cohort (9.9 mean years)	63154 (age 45–74)	Male, female	Quartile of BCX intake	Incidence of hip fracture	Yes	Intake of BCX was insignificant in reducing the risk of hip fracture in both males and females.	Good
Sahni, 2009 (2), United States [22]	Yes	Prospective cohort (17 years)	929 (age 75 ± 5)	Male, pre-/postmenopausal female	Tertile of BCX intake	Hazard ratios for hip fracture	Yes	There was an absence of evidence that the intake of BCX plays a role in the reduction of the risk of hip fracture.	Fair
Liu, 2018, China [23]	Yes	Case-control	Case, 196 (age 44.0–60.0); control, 196 (age 43.2–60.0)	Male, pre-/postmenopausal female	Quartile of BCX intake (mean value): Q1 (0.0762 mg/day) Q2 (0.1589 mg/day) Q3 (0.3139 mg/day) Q4 (0.8341 mg/day)	Odds ratios for skeletal fluorosis	Yes	Intake of BCX was ineffective in reducing the risk of skeletal fluorosis.	Fair
Cao, 2018, China [24]	Yes	Case-control	Case, 1070 (age 52–83); control, 1070 (age 52–83)	Male, postmenopausal female	Quartile of BCX intake (mean value): Q1 (0.030–0.031 mg/day) Q2 (0.063–0.064 mg/day) Q3 (0.096–0.097 mg/day) Q4 (0.151–0.164 mg/day)	Odds ratios for hip fracture	Yes	The highest consumption quartile of BCX showed significantly reduced odd ratios on hip fractures. Subgroup analysis by gender revealed a corresponding result in terms of reducing hip fracture risk.	Good
Zhang, 2016, China [25]	No	Prospective cohort (3.1 mean years)	2831 (age 50–75)	Male, postmenopausal female	Quartile of serum BCX concentration	BMD of the whole body, hip (total), femur neck, trochanter, intertrochanter	Yes	The serum level of BCX was positively correlated to the higher value of lumber spine and femur neck BMD in postmenopausal women.	Fair
Sugiura, 2016, Japan [26]	No	Prospective cohort (4 years)	187 (age 60.5 ± 5.8)	Postmenopausal female	Low (0.24–1.84 μM) or high (1.88–10.53 μM) level of serum BCX concentration	Odds ratios for osteoporosis	Yes	The higher serum concentration of BCX with a higher intake of Vitamin C showed a significantly reduced risk of osteoporosis compared to the lower serum concentration of BCX with a low intake of vitamin C.	Fair
Sugiura, 2012, Japan [27]	No	Prospective cohort (4 years)	187 (age 60.5 ± 5.8)	Postmenopausal female	Tertile of serum BCX concentration Q1 (0.24–1.41 μM) Q2 (1.43–2.39 μM) Q3 (2.41–10.53 μM)	Odds ratios for osteoporosis and osteopenia	Yes	The higher serum concentration of BCX was significantly related to the reduced risk of osteopenia and osteoporosis.	Good
Imagama, 2011, Japan [28]	No	Cross-sectional	286 (age 50–85)	Male, female	Serum cryptoxanthin concentration (mean ± SD) Case, 0.25 ± 0.16 μM; control, 0.35 ± 0.33 μM	Existence of lumbar osteophyte	Not reported	The serum level of cryptoxanthin was significantly higher in ‘no osteophytes’ group.	Fair
Sahni. 2009 (1), United States [29]	No	Prospective cohort (4 years)	874 (age 75 ± 5)	Male, pre-/postmenopausal female	Tertile of BCX intake	Femur BMD; spine BMD; radius BMD	Yes	The intake of BCX was not related to the changes in any type of BMD.	Fair
Sugiura, 2008, Japan [30]	No	Cross-sectional	699 (age 30–70)	Male, pre-/postmenopausal female	Quartile of serum BCX concentration Q1 (0.22–1.07 mM) Q2–4 (1.10–10.53 mM)	Correlation analysis; odds ratios for low level of BMD	Yes	Positive correlations between serum BCX and BMD were observed in the postmenopausal female.	Fair
Yang, 2008, United States [31]	No	Case-control	Case, 30 (age 63.3 ± 10.8); control, 29 (age 62.1 ± 6.2)	Postmenopausal female	Serum BCX concentration (mean ± SD) Case, 0.27 ± 0.18 μM; control, 0.43 ± 0.36 μM Dietary BCX intake (mean ± SD) Case, 0.16 ± 0.19 mg/day; control, 0.09 ± 0.15 mg/day	Incidence of osteoporosis	Yes	The average serum concentration of BCX was significantly high in the control group compared to the osteoporosis group. Dietary BCX showed an opposite aspect as the overall intake of BCX was higher in the osteoporosis group.	Fair
Maggio, 2006, Italy [32]	No	Case-control	Case, 45 (age 70.3 ± 6.4); control, 45 (age 70.1 ± 5.9)	Postmenopausal female	Plasma BCX concentration Case, μM; 0.230 (0.129–0.397); control, 0.580 (0.363–0.715) μM	Incidence of osteoporosis	Not reported	The plasma concentration of BCX was significantly higher in control compared to the osteoporotic group.	Fair

†: Evaluated by following either Quality Assessment Tool for Observational Cohort and Cross-Sectional Studies or Quality Assessment of Case-Control Studies guideline of National Institutes of Health (NIH). BMD, bone mineral density; BMI, body mass index, BCX, *β*-cryptoxanthin.

## Supplementary Materials

The following are available online at https://www.mdpi.com/2304-8158/10/2/296/s1, Table S1: The Guidelines of Meta-analyses Of Observational Studies in Epidemiology (MOOSE) Checklist, Table S2: Quality assessment table of the included cohort and cross-sectional studies, Table S3: Quality assessment table of the included case-control studies, Table S4: Details of variable adjustment factors of the included studies.
