# Supplementary material for: Effects of β-Cryptoxanthin on Improvement in Osteoporosis Risk: A Systematic Review and Meta-Analysis of Observational Studies
Source: Foods. 2021 Feb 2;10(2):296. doi: 10.3390/foods10020296 (PMC7913073; doi:10.3390/foods10020296)
Supplement: Supplementary file 1 [file foods-10-00296-s001.pdf]

foods

Article

Effects of *β*-cryptoxanthin on improvement in osteoporosis risk: a systematic review and meta-analysis of observational studies

Sun Jo Kim[1,#], Nguyen Hoang Anh[1,#], Nguyen Co Diem[2], Seongoh Park[3], Young Hyun Cho[4], Nguyen Phuoc Long[5], In Guk Hwang[6], Johan Lim[4], Sung Won Kwon[1,5,*]

[1]College of Pharmacy, Seoul National University, Seoul, 08826, Republic of Korea; SJK (danielkim27@snu.ac.kr), NHA (2018-23140@snu.ac.kr), SKW (swkwon@snu.ac.kr)

[2]School of Medicine, Vietnam National University, Ho Chi Minh City 70000, Vietnam; NCD (ncdiem.stu15@medvnu.edu.vn)

[3]Department of Statistics, Sungshin Women's University, Seoul 08826, Republic of Korea; SP (spark6@sungshin.ac.kr)

[4]Department of Statistics, Seoul National University, Seoul 08826, Republic of Korea; YHC (yhjo93@snu.ac.kr), JL (johanlim@snu.ac.kr)

[5]Research Institute of Pharmaceutical Sciences, Seoul National University, Seoul, 08826, Republic of Korea; NPL (phuoclong@snu.ac.kr)

[6]Researcher, Department of Agrofood Resources, National Institute of Agricultural Sciences, Rural Development Administration, Wanju 55365, Republic of Korea; IGH (ighwang79@korea.kr)

[#]Authors contributed equally

* Correspondence: S.W.K. (swkwon@snu.ac.kr);

Table S2. NIH quality assessment of cohort and cross-sectional studies.

Study	Item 1	Item 2	Item 3	Item 4	Item 5	Item 6	Item 7	Item 8	Item 9	Item 10	Item 11	Item 12	Item 13	Item 14	Quality Rating (Good, Fair, or Poor)
Regu, 2017 [1]	Yes	Yes	Yes	Yes	No	NR	CD	Yes	No	No	Yes	NR	NA	Yes	Fair
Sugiura, 2008 [2]	Yes	Yes	Yes	Yes	No	NR	No	Yes	No	NR	Yes	NR	NA	Yes	Fair
Hayhoe, 2017 [3]	Yes	Yes	Yes	Yes	No	Yes	Yes	Yes	Yes	No	Yes	NR	No	Yes	Good
Sugiura, 2011 [4]	Yes	Yes	Yes	Yes	No	NR	No	Yes	No	NR	Yes	NR	Yes	Yes	Fair
Zhang, 2016 [5]	Yes	Yes	Yes	No	No	Yes	Yes	Yes	Yes	NR	Yes	NR	No	Yes	Fair
Sugiura, 2012 [6]	Yes	Yes	Yes	Yes	No	Yes	Yes	Yes	Yes	No	Yes	NR	No	Yes	Good
Sugiura, 2016 [7]	Yes	Yes	NR	Yes	No	Yes	Yes	Yes	Yes	No	Yes	NR	Yes	Yes	Fair
Sahni, 2009 (1) [8]	Yes	Yes	Yes	Yes	No	Yes	Yes	Yes	No	No	Yes	NR	No	Yes	Fair
Sahni, 2009 (2) [9]	Yes	Yes	Yes	Yes	No	Yes	Yes	Yes	No	No	CD	NR	Yes	Yes	Fair
Imagama, 2011 [10]	Yes	Yes	No	No	No	NR	No	No	Yes	No	Yes	Yes	NA	Yes	Fair
Dai, 2014 [11]	Yes	Yes	Yes	Yes	No	Yes	Yes	Yes	CD	No	Yes	NR	Yes	Yes	Good

Item 1: Was the research question or objective in this paper clearly stated?
Item 2: Was the study population clearly specified and defined?
Item 3: Was the participation rate of eligible persons at least 50%?
Item 4: Were all the subjects selected or recruited from the same or similar populations (including the same time period)? Were inclusion and exclusion criteria for being in the study prespecified and applied uniformly to all participants?
Item 5: Was a sample size justification, power description, or variance and effect estimates provided?
Item 6: For the analyses in this paper, were the exposure(s) of interest measured prior to the outcome(s) being measured?
Item 7: Was the timeframe sufficient so that one could reasonably expect to see an association between exposure and outcome if it existed?
Item 8: For exposures that can vary in amount or level, did the study examine different levels of the exposure as related to the outcome (e.g., categories of exposure, or exposure measured as continuous variable)?
Item 9: Were the exposure measures (independent variables) clearly defined, valid, reliable, and implemented consistently across all study participants?
Item 10: Was the exposure(s) assessed more than once over time?
Item 11: Were the outcome measures (dependent variables) clearly defined, valid, reliable, and implemented consistently across all study participants?
Item 12: Were the outcome assessors blinded to the exposure status of participants?
Item 13: Was loss to follow-up after baseline 20% or less?
Item 14: Were key potential confounding variables measured and adjusted statistically for their impact on the relationship between exposure(s) and outcome(s)?
Yes; No; CD, cannot determine; NA, not applicable; NR, not reported

Table S3. NIH quality assessment of case-control studies.

Study	Item 1	Item 2	Item 3	Item 4	Item 5	Item 6	Item 7	Item 8	Item 9	Item 10	Item 11	Item 12	Quality Rating (Good, Fair, or Poor)
Liu, 2018 [12]	Yes	Yes	No	Yes	Yes	Yes	NR	NR	No	Yes	NR	Yes	Fair
Cao, 2018 [13]	Yes	Yes	Yes	Yes	Yes	Yes	NR	NR	NR	Yes	NR	Yes	Good
Maggio, 2006 [14]	Yes	Yes	No	Yes	Yes	Yes	NR	NR	No	Yes	Yes	No	Fair
Yang, 2008 [15]	Yes	Yes	No	Yes	Yes	Yes	NR	NR	NR	Yes	NR	Yes	Fair

Item 1: Was the research question or objective in this paper clearly stated and appropriate?
Item 2: Was the study population clearly specified and defined?
Item 3: Did the authors include a sample size justification?
Item 4: Were controls selected or recruited from the same or similar population that gave rise to the cases (including the same timeframe)?
Item 5: Were the definitions, inclusion and exclusion criteria, algorithms or processes used to identify or select cases and controls valid, reliable, and implemented consistently across all study participants?
Item 6: Were the cases clearly defined and differentiated from controls?
Item 7: If less than 100 percent of eligible cases and/or controls were selected for the study, were the cases and/or controls randomly selected from those eligible?
Item 8: Was there use of concurrent controls?
Item 9: Were the investigators able to confirm that the exposure/risk occurred prior to the development of the condition or event that defined a participant as a case?
Item 10: Were the measures of exposure/risk clearly defined, valid, reliable, and implemented consistently (including the same time period) across all study participants?
Item 11: Were the assessors of exposure/risk blinded to the case or control status of participants?
Item 12: Were key potential confounding variables measured and adjusted statistically in the analyses? If matching was used, did the investigators account for matching during study analysis?
Yes; No; CD, cannot determine; NA, not applicable; NR, not reported

Table S4. Detail of variable adjustment factors of the included studies.

Author, year, location	Included to meta-analysis	Study design	Adjustment variable
Regu, 2017, South Korea [1]	Yes	Cross-sectional	Model 1: age, BMI; model 2: age, BMI, alcohol consumption, physical activity, education level, supplement use, energy-adjusted intake of fiber, vitamin C, calcium, and sodium, and serum 25(OH)D
Sugiura, 2011, Japan [4]	Yes	Cross-sectional	Model 1: age, weight, height; model 2: age, weight, height, current tobacco use, years since menopause, regular alcohol intake, exercise habits, supplement use, total energy intake; model 3: age, weight, height, current tobacco use, years since menopause, regular alcohol intake, exercise habits, supplement use, total energy intake, calcium, magnesium, potassium, vitamin D
Hayhoe, 2017, United Kingdom [3]	Yes	Prospective cohort (12.5 mean years)	Model for intake: age, BMI, family history of osteoporosis, menopausal and hormone replacement therapy status in women, corticosteroid use, smoking status, physical activity, Ca intake, total energy intake, Ca- and vitamin D-containing supplement use, days of food diary completed and the ratio of energy intake; a model for serum level: age, BMI, family history of osteoporosis, menopausal and hormone replacement therapy status in women, corticosteroid use, smoking status, physical activity
Dai, 2014, Singapore [11]	Yes	Prospective cohort (9.9 mean years)	Age, recruitment year, dialect group, body mass index, level of education in categories, total energy intake, smoking status, physical activity, calcium, soy isoflavones, vitamin B6, menopausal status, use of hormone replacement therapy at recruitment, baseline physician-diagnosed history of diabetes and stroke
Sahni, 2009 (2), United States [9]	Yes	Prospective cohort (17 years)	Sex, age, BMI, height, energy intake, physical activity, alcohol intake, smoking, calcium intake, vitamin D intake, caffeine intake
Liu, 2018, China [12]	Yes	Case-control	Model 1: no adjustment; model 2: energy intake, marital status, education level, smoking, alcohol drinking, tea-drinking, fuel type, improved stove use, roasting food, roasted chilli consumption; model 3: energy intake, marital status, education level, smoking, alcohol drinking, tea-drinking, fuel type, improved stove use, roasting food, roasted chilli consumption, calcium intake, rs11968525 genotype, urinary fluoride level
Cao, 2018, China [13]	Yes	Case-control	Model 1: age, sex, BMI; model 2: age, sex, BMI, occupation, marital status, educational level, household income, current smoking, passive smoking, current drinking, calcium and multivitamin supplement, family history of fractures, physical activity, daily energy and calcium intake; model 3: age, sex, BMI, occupation, marital status, educational level,

			household income, current smoking, passive smoking, current drinking, calcium and multivitamin supplement, family history of fractures, physical activity, daily energy, and calcium intake, dietary potassium, magnesium, caffeine, net endogenous acid production, potential renal acid load, and Alternative Mediterranean diet score.
Zhang, 2016, China [5]	No	Prospective cohort (3.1 mean years)	Age, BMI, household income, smoking status, alcohol status, calcium supplement use, multivitamin use, dietary intake of energy, protein, calcium, potassium, and magnesium, physical activity, the season for blood sample collection, estrogen use, years since menopause (only for women)
Sugiura, 2016, Japan [7]	No	Prospective cohort (4 years)	Age, weight, height, years since menopause, current tobacco use, regular alcohol intake, exercise habits, supplement use, total energy intake
Sugiura, 2012, Japan [6]	No	Prospective cohort (4 years)	Model 1: age, weight, height, years since menopause, current tobacco use, regular alcohol intake, exercise habits, supplement use, total energy intake; model 2: age, weight, height, years since menopause, current tobacco use, regular alcohol intake, exercise habits, supplement use, total energy intake, calcium, magnesium, potassium, vitamins D, C, and E
Imagama, 2011, Japan [10]	No	Cross-sectional	Not reported
Sahni. 2009 (1), United States [8]	No	Prospective cohort (4 years)	Age, BMI, height, physical activity index, smoking (never compared with ever smokers), multivitamin use, a season of BMD measurement (for cross-sectional analyses on BMD only), estrogen use (in women), and intakes of total energy, calcium, vitamin D, caffeine, and alcohol
Sugiura, 2008, Japan [2]	No	Cross-sectional	Model 1: age, weight, height; model 2: age, weight, height, current tobacco use, regular alcohol intake, exercise habits, supplement use, total energy intake; model 3: age, weight, height, current tobacco use, regular alcohol intake, exercise habits, supplement use, and intake of total energy, calcium, magnesium, potassium, vitamins D, C, and E
Yang, 2008, United States [15]	No	Case-control	Serum BCX model 1: no adjustment; serum BCX model 2: age, BMI; dietary BCX: age, BMI, supplement use, fruit and vegetable consumption, milk intake
Maggio, 2006, Italy [14]	No	Case-control	Not reported

BMD: Bone mineral density; BMI: body mass index, BCX: β-cryptoxanthin

References

1. Regu, G.M.; Kim, H.; Kim, Y.J.; Paek, J.E.; Lee, G.; Chang, N.; Kwon, O. Association between Dietary Carotenoid Intake and Bone Mineral Density in Korean Adults Aged 30-75 Years Using Data from the Fourth and Fifth Korean National Health and Nutrition Examination Surveys (2008-2011). *Nutrients* **2017**, *9*, doi:10.3390/nu9091025.
2. Sugiura, M.; Nakamura, M.; Ogawa, K.; Ikoma, Y.; Ando, F.; Yano, M. Bone mineral density in post-menopausal female subjects is associated with serum antioxidant carotenoids. *Osteoporosis international : a journal established as result of cooperation between the European Foundation for Osteoporosis and the National Osteoporosis Foundation of the USA* **2008**, *19*, 211-219, doi:10.1007/s00198-007-0457-2.
3. Hayhoe, R.P.G.; Lentjes, M.A.H.; Mulligan, A.A.; Luben, R.N.; Khaw, K.T.; Welch, A.A. Carotenoid dietary intakes and plasma concentrations are associated with heel bone ultrasound attenuation and osteoporotic fracture risk in the European Prospective Investigation into Cancer and Nutrition (EPIC)-Norfolk cohort. *The British journal of nutrition* **2017**, *117*, 1439-1453, doi:10.1017/s0007114517001180.
4. Sugiura, M.; Nakamura, M.; Ogawa, K.; Ikoma, Y.; Ando, F.; Shimokata, H.; Yano, M. Dietary patterns of antioxidant vitamin and carotenoid intake associated with bone mineral density: findings from post-menopausal Japanese female subjects. *Osteoporosis international : a journal established as result of cooperation between the European Foundation for Osteoporosis and the National Osteoporosis Foundation of the USA* **2011**, *22*, 143-152, doi:10.1007/s00198-010-1239-9.
5. Zhang, Z.Q.; Cao, W.T.; Liu, J.; Cao, Y.; Su, Y.X.; Chen, Y.M. Greater serum carotenoid concentration associated with higher bone mineral density in Chinese adults. *Osteoporosis international : a journal established as result of cooperation between the European Foundation for Osteoporosis and the National Osteoporosis Foundation of the USA* **2016**, *27*, 1593-1601, doi:10.1007/s00198-015-3425-2.
6. Sugiura, M.; Nakamura, M.; Ogawa, K.; Ikoma, Y.; Yano, M. High serum carotenoids associated with lower risk for bone loss and osteoporosis in post-menopausal Japanese female subjects: prospective cohort study. *PLoS One* **2012**, *7*, e52643-e52643, doi:10.1371/journal.pone.0052643.
7. Sugiura, M.; Nakamura, M.; Ogawa, K.; Ikoma, Y.; Yano, M. High Vitamin C Intake with High Serum β-Cryptoxanthin Associated with Lower Risk for Osteoporosis in Post-Menopausal Japanese Female Subjects: Mikkabi Cohort Study. *Journal of nutritional science and vitaminology* **2016**, *62*, 185-191, doi:10.3177/jnsv.62.185.
8. Sahni, S.; Hannan, M.T.; Blumberg, J.; Cupples, L.A.; Kiel, D.P.; Tucker, K.L. Inverse association of carotenoid intakes with 4-y change in bone mineral density in elderly men and women: the Framingham Osteoporosis Study. *The American journal of clinical nutrition* **2009**, *89*, 416-424, doi:10.3945/ajcn.2008.26388.

9. Sahni, S.; Hannan, M.T.; Blumberg, J.; Cupples, L.A.; Kiel, D.P.; Tucker, K.L. Protective effect of total carotenoid and lycopene intake on the risk of hip fracture: a 17-year follow-up from the Framingham Osteoporosis Study. *J Bone Miner Res* **2009**, *24*, 1086-1094, doi:10.1359/jbmr.090102.
10. Imagama, S.; Hasegawa, Y.; Seki, T.; Matsuyama, Y.; Sakai, Y.; Ito, Z.; Ishiguro, N.; Ito, Y.; Hamajima, N.; Suzuki, K. The effect of β-carotene on lumbar osteophyte formation. *Spine* **2011**, *36*, 2293-2298, doi:10.1097/BRS.0b013e3182254a18.
11. Dai, Z.; Wang, R.; Ang, L.W.; Low, Y.L.; Yuan, J.M.; Koh, W.P. Protective effects of dietary carotenoids on risk of hip fracture in men: the Singapore Chinese Health Study. *J Bone Miner Res* **2014**, *29*, 408-417, doi:10.1002/jbmr.2041.
12. Liu, J.; Yang, S.; Luo, M.J.; Zhao, X.; Zhang, Y.M.; Luo, Y. Association of Dietary Carotenoids Intake with Skeletal Fluorosis in the Coal-burning Fluorosis Area of Guizhou Province. *Biomedical and environmental sciences : BES* **2018**, *31*, 438-447, doi:10.3967/bes2018.057.
13. Cao, W.T.; Zeng, F.F.; Li, B.L.; Lin, J.S.; Liang, Y.Y.; Chen, Y.M. Higher dietary carotenoid intake associated with lower risk of hip fracture in middle-aged and elderly Chinese: A matched case-control study. *Bone* **2018**, *111*, 116-122, doi:10.1016/j.bone.2018.03.023.
14. Maggio, D.; Polidori, M.C.; Barabani, M.; Tufi, A.; Ruggiero, C.; Cecchetti, R.; Aisa, M.C.; Stahl, W.; Cherubini, A. Low levels of carotenoids and retinol in involutional osteoporosis. *Bone* **2006**, *38*, 244-248, doi:10.1016/j.bone.2005.08.003.
15. Yang, Z.; Zhang, Z.; Penniston, K.L.; Binkley, N.; Tanumihardjo, S.A. Serum carotenoid concentrations in postmenopausal women from the United States with and without osteoporosis. *Int J Vitam Nutr Res* **2008**, *78*, 105-111, doi:10.1024/0300-9831.78.3.105.
